# From awareness to action: enhancing mothers’ knowledge and practices on lead exposure among young children in the Bani-Khaled Village, Minia Governorate

**DOI:** 10.1186/s12889-025-25961-6

**Published:** 2026-01-24

**Authors:** Mohammed Ibrahim Touni, Amal Mohamed Hashem, Manal Farouk Moustafa, Eman Sayed Ahmed, Heba Mostafa Mohammed, Essam Eltantawy Elsayed

**Affiliations:** 1 PhD in Community Health Nursing, Minia University Hospitals, Minia University, Minia, Egypt; 2 Community Health Nursing Department, College of Nursing, Jouf University, Sakaka, 72388, Saudi Arabia; 3 Nursing Department, North Private College of Nursing, Arar City, Northern Borders Saudi Arabia; 4 Community Health Nursing Department, Faculty of Nursing, Minia University, Minia City, Egypt; 5 Maternity and Newborn Health Nursing, Dean of Faculty of Nursing, Badr University in Assiut University, Assiut City, Egypt; 6 Child and adolescent health nursing faculty of Nursing Faculty of Nursing, Badr University in Assiut, Assiut City, Egypt; 7 Maternal and Newborn Health Nursing, Faculty of Nursing, Al-Ahliyya Amman University, Amman, Jordan; 8 Department of Nursing, College of nursing Sciences, Hafr Albatin University, Hafr Albatin, Saudi Arabia

**Keywords:** Mothers’ knowledge, Practices, Lead exposure, Young children, Sustainability

## Abstract

**Background:**

Children’s health is a basic pillar for sustainable development, directly influencing social stability, economic growth, and national progress. Lead exposure represents a community health risk, particularly among children. High amounts of lead exposure may have negative effects on the brain, learning, and central nervous system and may result in coma, death, and seizures.

The aim of the current study was to evaluate the effectiveness of an educational intervention in enhancing mothers’ knowledge and practices regarding lead exposure among young children in Bani-Khaled Village, Minia Governorate.

**Methods:**

A quasi experimental design was utilized to assess mothers’ knowledge and practices pre- and postintervention. The intervention was divided into eight sessions, lectures, group discussions, brainstorming, movies, and photos. A systematic random sample of 251 mothers was selected. Two hundred fifty-one mothers participated. Data analysis was carried out via descriptive statistics, chi-square tests, paired t tests, Pearson’s correlation, and multiple regression techniques.

**Results:**

Postintervention evaluation demonstrated the educational intervention was associated with notable improvements in mothers’ knowledge and practices regarding lead exposure among young children in Bani-Khaled village. The mean knowledge score increased from 8.5 ± 2.3 to 15.2 ± 2.1 (t = 38.47, *p* < 0.001). While the mean practice score increased from 9.2 ± 1.8 to 14.3 ± 1.9 (t = 39.12, *p* < 0.001). While a strong correlation was observed between knowledge and practices before the intervention, the post-intervention correlation was weak.

**Conclusion:**

The educational intervention demonstrated a positive effect on improving mothers’ knowledge and practices regarding lead exposure among young children. Future research should incorporate longer follow-up periods and broader community samples to better understand program effectiveness across different contexts and caregiver groups.

## Introduction

Children’s health is a basic pillar for sustainable development, directly influencing social stability, economic growth, and national progress. Children are vital to the nation’s present and future, so children represent an important sector of the population that is constantly growing and developing [1]. This basic dynamic characteristic accounts for their increased vitality and vulnerability and requires specific health promotion in relation to seeking health and using various resources to attain optimum health. Young children are particularly vulnerable to the toxic effects of lead and can suffer permanent adverse health impacts, particularly on the development of the central nervous system [2].

Lead exposure represents a community health risk, particularly among children. High amounts of lead exposure may have dangerous effects on the brain, learning, and central nervous system, kidneys, blood, and cardiovascular system, as children grow and develop as well as coma, death, and seizures [2]. The presence of lead in food, water, paint, air, soil, toys, cosmetics and medicines could not be detected with naked eye. In children, elevated blood lead levels are among the leading public health issues worldwide [3, 4].

Lead is distributed throughout organs such as the brain, kidneys, liver, and bones once it enters the body. It is largely stored in bones and teeth for extended periods. During pregnancy, this reservoir may be released into the blood, endangering fetal development. Nutrient-poor children, particularly those lacking calcium or iron, are at greater risk due to increased absorption [5, 6].

Children could be exposed to lead exposure by inhaling or ingesting contaminated soil while playing outdoors or when lead particles are carried indoors on shoes or clothing. Exposure can also occur through dust originating from soil polluted by sources such as leaded gasoline, aviation fuel, mining activities, and industrial operations [7]. Drinking water through lead-containing pipes, faucets, or plumbing fixtures is another common route. Additionally, certain cosmetics and traditional remedies—such as azarcon and greta, which are often used in Hispanic communities for digestive issues—may contain lead. Other sources include some candies and their packaging, consumer goods such as toys, jewelry, antiques, and lead-glazed ceramics [8–10].

Mothers should use positive strategies to prevent disease and promote the health of their children [11]. Poor awareness among mothers is recognized as a major risk factor for accidental poisoning in children. A descriptive study conducted in Egypt assessed the knowledge and health habits of mothers regarding lead pollution. The findings revealed that while over two-fifths of mothers demonstrated good knowledge, more than half had poor understanding of lead sources and health consequences. Moreover, a statistically significant positive correlation was found between maternal knowledge and preventive practices [12]. In Bangladesh, caregivers showed limited knowledge about lead risks. Despite moderate risk perception, protective practices were inconsistently applied, indicating that awareness directly influences caregivers’ practices [13]. Additionally, a cross-sectional study in Cairo revealed that children exposed to lead through environmental and occupational sources and emphasized the need for national prevention programs and establishment of mass lead education that targets parents to increase the awareness of the problem and altering the attitudes related to prevention [14].

Poisoning is considered the critical leading cause of death in Egypt, especially among children, and is a critical area for prevention and awareness efforts [15]. Young children are particularly vulnerable to lead poisoning, as they may absorb up to 4–5 times as much lead as adults from an ingested dose. A lack of mothers’ awareness is an identified risk factor for unintentional child poisoning; thus, identifying the level of awareness and existing gaps among mothers is essential, as this information can guide the development of effective prevention strategies [16]. Therefore, this study aims to evaluate the effectiveness of an educational intervention in enhancing mothers’ knowledge and practices regarding lead exposure among young children in Bani-Khaled Village, Minia Governorate. Specifically, the study seeks to: (1) assess baseline knowledge and practices of mothers toward lead exposure; (2) implement a structured educational intervention; and (3) measure changes in knowledge and practices post-intervention. We hypothesize that mothers who receive the educational intervention will demonstrate improvement in their knowledge and practices compared to their baseline scores.

## Methods

### Study design

This study used a quasiexperimental design to evaluate the effectiveness of an educational intervention pre- and postintervention.

### Setting

The study was carried out in Bani-Khaled Village, which is located in the Samalout District of Minia Governorate, Egypt. The village is known for environmental exposure risks due to proximity to industrial activities.

### Participants

A systematic random sample of 251 mothers was selected from Bani-Khaled Village. The sample size was determined using the standard single-population proportion formula:

$$\frac{Z^2\;\times\;P(1-P)}{2_d}\;=\;n$$

where *Z* represents the standard normal value at a 95% confidence level (1.96), *P* denotes the expected proportion of mothers with adequate knowledge regarding lead exposure (assumed to be 0.50 due to the absence of prior local evidence), and *d* refers to the acceptable margin of error (0.05). The calculation yielded a minimum required sample size of 246 mothers. To compensate for potential non-response, an additional 5–10% was considered, resulting in a final sample size of 251 participants. The inclusion criteria were mothers of children aged 0–12 years, residents of the Bani-Khaled Village and willing to participate and provide informed consent.

### Intervention procedure

The pretest was performed via a questionnaire. On the basis of the results of the pretest, the educational materials, methods and number of sessions needed for education were determined. Educational content was tailored to the mothers’ level of understanding and developed via credible scientific sources. The intervention plan included eight sessions over a two-month period organized into groups of 10–15 participants. Each session lasted 50–60 min, with a 10-minute break. The teaching strategies included lectures, group discussions, brainstorming, and visual aids such as films and photographs. All the participants attended every session. The program also featured a one-hour session focused on lead poisoning, covering its sources, exposure pathways, health effects, and prevention. A posttest was conducted three months later to evaluate changes in knowledge and practices following the intervention.

The educational session addressed key aspects of lead exposure, beginning with an overview of its definition, prevalence, and recent statistics relevant to rural Egyptian communities. This study highlighted major environmental and household sources of lead exposure in Bani-Khaled village and explained the primary routes of exposure for young children, alongside common high-risk practices in rural settings. The session emphasized the physical, cognitive, and social benefits of preventing lead exposure, particularly during early childhood, and discussed the challenges and barriers to adopting lead-safe behaviors. Strategies to increase mothers’ self-efficacy in protecting their children from lead hazards were also introduced.

### Data collection tools

A structured questionnaire was adapted from a previously published study that assessed mothers’ knowledge and health habits regarding lead exposure in Bani-Khaled Village, Minia Governorate. The original tool was developed by Ismael et al. [13] and was modified to align with the objectives and intervention components of the current research. It includes two instruments.

The first instrument gathered mothers’ demographic details and mothers’ knowledge about lead exposure. It includes 6 questions, designed to gather demographic data (age, education level, occupation, monthly household income, number of children, and source of health information) and 38 question designed to assess mothers’ understanding of lead exposure, lead poisoning, and common household causes of lead exposure [12, 17]. The knowledge of mothers was assessed via a binary scoring system, where each correct (‘yes’) response was given a score of 1, and incorrect or ‘don’t know’ responses were assigned a score of 0. This method was used to objectively differentiate between correct and incorrect knowledge responses, as commonly applied in similar intervention studies. The total knowledge score was calculated by summing all the items, yielding a maximum possible score of 38. Mothers who achieved ≥ 50% of the total score (≥ 19 out of 38) were categorized as having good knowledge, whereas those scoring below 50% (< 19 out of 38) were classified as having poor knowledge [12, 18].

The second instrument contains 18 questions assessing the practices of mothers with respect to lead exposure, such as the use of cosmetics, hair dyes, etc.), with answers of yes or no [18]. The scoring system of this tool varied from zero for no and one for yes. The maximum score is [18]. Mothers were considered to have a satisfactory level of practice if their total score was ≥ 50% (≥ 9 out of 18). Conversely, a score of less than 50% (< 9 out of 18) was classified as an unsatisfactory level of practice [12, 18].

### Validity and reliability

The content validity of the data collection instruments was established through an extensive review of the literature on lead contamination. The tools were subsequently evaluated by a panel of five experts specializing in community health nursing, public health, and preventive medicine. On the basis of their feedback regarding item relevance, sequencing, and clarity, necessary modifications were made to increase the accuracy and appropriateness of the instruments. To ensure reliability after these modifications, a pilot study was conducted on 25 mothers (representing 10% of the total sample) who were excluded from the main study. The internal consistency of the questionnaire was assessed using Cronbach’s alpha coefficient, which was 0.87 for the knowledge section and 0.81 for the practice section, indicating satisfactory reliability of the tool.

### Ethical considerations

Ethical approval was obtained from the Ethics Committee of the Faculty of Nursing, Badr University, in Assuit. Written informed consent was obtained from all participants. Strict confidentiality and anonymity were maintained throughout the study.

### Statistical analysis

The data were recorded, organized, and analyzed using the Statistical Package for the Social Sciences (SPSS), version 20. Descriptive statistics were applied, with qualitative data presented as frequencies and percentages, while quantitative data were summarized using means and standard deviations. The chi-square test was used to examine associations between categorical variables. The paired t-test was employed to compare mean knowledge and practice scores before and after the intervention within the same group. Pearson’s correlation coefficient was used to assess the relationship between knowledge and practices. Multiple linear regression analysis was conducted to identify the predictors of mothers’ post-intervention knowledge and practice scores. A *p*-value of ≤ 0.05 was considered statistically significant.

## Results

Table (1) shows that 44.6% of the participants were aged between 25 and 34 years, whereas 33.9% were older than 35 years, with a mean age of 31 ± 7.5 years. With respect to monthly family income, 66.9% of mothers had income < 3000 EGP, whereas 33.1% had income ≥ 3000 EGP. With respect to mothers’ educational level, 37.8% had primary education, and 31.1% were illiterate. Most of the studied mothers (36.3%) reported that their primary source of knowledge about lead exposure was TV/radio, whereas 24.7% obtained their knowledge from relatives/neighbors.

**Table 1:** Demographic characteristics of the participating mothers (*n* = 251)

Variable	Category	*n*	%
Age	< 25	54	21.5
	25–34	112	44.6
	≥ 35	85	33.9
Mean	31 ± 7.5		
Education Level	Illiterate	78	31.1
	Primary	95	37.8
	Secondary and above	78	31.1
Monthly Household Income	< 3000 EGP	168	66.9
	≥ 3000 EGP	83	33.1
Source of Health Info	TV/radio	91	36.3
	Health unit	58	23.1
	Relatives/neighbors	62	24.7
	Social media	40	15.9

Table (2) shows that 55.7% of the studied mothers had poor knowledge regarding lead exposure before the educational intervention. This improved after the intervention, with 87.7% demonstrating good knowledge. The mean knowledge score increased from 8.5 ± 2.3 before the intervention to 15.2 ± 2.1 after it. There were highly statistically significant differences in mothers’ knowledge before and after the educational intervention (*p* value ≤ 0.001).

**Table 2:** Distribution of mothers by overall knowledge scores on lead exposure pre- and postintervention

Mothers’ Knowledge	Pre		Post		*χ*2	*P* value
	No	%	No	%		
Good	111	44.3	220	87.7%	437.263	0.001
Poor	140	55.7	31	12.3		
Mean	8.5 ± 2.3		15.2 ± 2.1			

Table (3) shows that 78.1% of the studied mothers had satisfactory practices regarding lead exposure before the educational intervention, whereas 90.3% had satisfactory practices after the intervention. The mean practice score increased from 9.2 ± 1.8 before the intervention to 14.3 ± 1.9 afterward. There was a highly statistically significant difference (*p* value ≤ 0.001).

**Table 3:** Mothers’ overall practice scores for lead exposure before and after the educational intervention

Mothers’ practices	Pre		Post		χ^2^	*P* value
Satisfactory	196	78.1	227	90.3	44.275	0.001
Un satisfactory	55	21.9	24	9.7		
Mean	9.2 ± 1.8		14.3 ± 1.9			

Table (4) shows postintervention is associated with improvement in both knowledge and practice scores post-intervention. The mean knowledge score increased from 8.5 ± 2.3 to 15.2 ± 2.1 (t = 38.47, *p* < 0.001), while the mean practice score increased from 9.2 ± 1.8 to 14.3 ± 1.9 (t = 39.12, *p* < 0.001).

**Table 4:** Comparison of mothers’ knowledge and practice scores related to lead exposure among young children before and after the educational intervention using Paired t-test

Variable	Mean ± SD (Pre)	Mean ± SD (Post)	t (df = 250)	*p*-value
Knowledge Score	8.5 ± 2.3	15.2 ± 2.1	38.47	< 0.001
Practice Score	9.2 ± 1.8	14.3 ± 1.9	39.12	< 0.001

Table (5) shows positive correlation between pre-intervention knowledge and pre-intervention practices (*r* = 0.960, *p* < 0.001). However, the correlation between post-intervention knowledge and post-intervention practices was weak but statistically significant (*r* = 0.077, *p* = 0.001).

**Table 5:** Correlations between overall scores of women’s knowledge of lead exposure among young children and their practices before and after educational intervention

			Knowledge (pre)	Knowledge (post)
Pre	Practices	*R*	0.960	
		*P* – value	0.001	
Post	Practices	*R*		0.077
		*P* – value		0.001

Table (6) reveals several factors that predict mothers’ postintervention knowledge scores about lead exposure. Mothers’ education level was the strongest predictor (β = 0.315, *p* < 0.001). Additionally, having a father employed in crafts involving lead (β = 0.189, *p* = 0.003) and sufficient monthly family income (β = 0.143, *p* = 0.025) were both associated with higher knowledge scores.

**Table 6:** Multiple regression analysis predicting mothers’ postintervention knowledge scores about lead exposure among young children

Predictor Variable	β (Standardized Coefficient)	SE	t	*p* value	95% Confidence Interval (CI)
Mother’s education (years)	0.315	0.061	5.16	< 0.001	[0.196, 0.434]
Father’s occupation in crafts with lead	0.189	0.049	3.01	0.003	[0.093, 0.285]
Monthly family income sufficiency (enough vs. not)	0.143	0.057	2.26	0.025	[0.031, 0.255]
Constant			4.02	< 0.001	

Table (7) shows that mothers’ postintervention practice scores regarding lead exposure are influenced by three factors. The knowledge score was a strong predictor postintervention (β = 0.266, *p* < 0.001). Additionally, mothers’ education had a strong effect (β = 0.233, *p* < 0.001). Mothers’ age has a weak significant effect (β = 0.119, *p* = 0.045).

**Table 7:** Multiple regression analysis predicting mothers’ postintervention practice scores for lead exposure

Predictor Variable	β (Standardized Coefficient)	SE	t	*p* value	95% Confidence Interval (CI)
Postintervention knowledge score	0.266	0.055	4.19	< 0.001	[0.158, 0.374]
Mother’s education (years)	0.233	0.052	3.56	< 0.001	[0.131, 0.335]
Age	0.119	0.031	2.01	0.045	[0.058, 0.180]
Constant	—	—	3.72	< 0.001	—

## Discussion

The present study revealed that more than half of the studied mothers had a low level of knowledge regarding lead exposure before the educational intervention, which improved after the intervention, and the majority demonstrated good knowledge, with highly statistically significant differences in mothers’ knowledge before and after the educational intervention. These results are in line with those of Avila [19], who assessed the impact of brief educational sessions provided by health care workers to parents in El Paso, Texas, and reported a significant increase in knowledge among parents with childhood lead exposure following the one-on-one education session offered by community health workers about lead. This result was also confirmed by Balza et al. [20], who reported that educational interventions may decrease the severity of lead poisoning in children. Similarly, Isenaj et al. [21] reported the effectiveness of educational interventions in enhancing awareness.

With respect to mothers’ practices regarding lead exposure, the current study revealed that the mean practice score increased from 9.2 ± 1.8 before the intervention to 14.3 ± 1.9 after the intervention, with a highly statistically significant difference. This may be because Egyptian communities often have limited access to health education and environmental health information. Mothers in rural areas are highly motivated to protect their children’s health. When provided with structured, culturally appropriate education, they tend to adopt new knowledge and practices quickly.

These findings are consistent with those of Sivabalan and Kale [22], who reported that the overall pretest score (12.1 ± 2.96) reflected an ‘average’ level of parental awareness regarding lead poisoning, whereas the posttest score (20.4 ± 2.61) indicated a ‘good’ level. The improvement was statistically significant at the *p* < 0.001. These results are also in accordance with those of Sprague et al. [23], who reported that educational programs lead to improved behaviors and practices related to environmental hazards.

With respect to the overall scores of women’s knowledge about lead exposure, the present study revealed that there was a positive correlation between pre-intervention knowledge and pre-intervention practices. However, the correlation between post-intervention knowledge and post-intervention practices was weak but statistically significant. These results are in line with those of Ismael et al. [12], who reported a positive significant correlation between mothers’ knowledge of lead exposure and their practices. Additionally, this result was confirmed by Omar et al. [24], who reported a significant positive correlation (*r* = 0.560, *p* < 0.001) between overall knowledge and reported practices after an educational intervention. In addition, these results are supported by Neta et al. [25], who confirmed that improved knowledge often translates into better practices.

The current study revealed that the mother’s education level was the strongest predictor (β = 0.315, *p* < 0.001). Additionally, having a father employed in crafts involving lead (β = 0.189, *p* = 0.003) and sufficient monthly family income (β = 0.143, *p* = 0.025) were both associated with higher knowledge scores. These results may be related to educated mothers being more likely to understand the risks and take preventive actions. These results are supported by a study in Bangladesh, which reported that mothers with more than five years of schooling had higher knowledge scores about lead exposure [13]. Additionally, these results were confirmed by Ismael et al. [12], who reported a positive correlation between mothers’ education levels and their knowledge and practices regarding lead exposure. A systematic review revealed that fathers working in lead-related industries may indirectly influence maternal awareness through shared household risks [26].

This study has several limitations. First, the research was conducted in a single rural setting (Bani-Khaled Village), which may restrict the generalizability of findings to other regions with different socioeconomic and cultural contexts. Second, although a systematic random sampling technique was employed, the sample was limited to mothers of children aged 0–12 years, which may not capture the perspectives of other caregivers. Third, the postintervention evaluation occurred three months after program implementation, which may not fully reflect long-term behavioral sustainability. Despite these limitations, the study offers evidence supporting the role of the educational intervention in mitigating lead exposure risks among vulnerable populations.

## Conclusion

The educational intervention demonstrated a positive effect on improving mothers’ knowledge and practices regarding lead exposure among young children. While the findings highlight the value of educational intervention in reducing environmental health risks, ongoing evaluation is needed to assess the long-term sustainability of behavioral change. These findings highlight the critical role of community-based health education in addressing environmental health risks, particularly in vulnerable populations where awareness is limited. Future research should incorporate longer follow-up periods and broader community samples to better understand program effectiveness across different contexts and caregiver groups.

## Data Availability

The datasets used and/or analyzed during the current study are available from the corresponding author on reasonable request.
